# Nestling odour modulates behavioural response in male, but not in female zebra finches

**DOI:** 10.1038/s41598-020-80466-z

**Published:** 2021-01-12

**Authors:** Sarah Golüke, Hans-Joachim Bischof, Barbara A. Caspers

**Affiliations:** 1 Department of Behavioural Ecology, Bielefeld University, Bielefeld, Germany; 2 Department of Animal Behaviour, Bielefeld University, Bielefeld, Germany

**Keywords:** Animal behaviour, Chemical ecology

## Abstract

Studies investigating parent offspring recognition in birds led to the conclusion that offspring recognition is absent at the early nestling stage. Especially male songbirds were often assumed to be unable to discriminate between own and foreign offspring. However, olfactory offspring recognition in birds has not been taken into account as yet, probably because particularly songbirds have for a long time been assumed anosmic. This study aimed to test whether offspring might be recognised via smell. We presented zebra finch (*Taeniopygia guttata*) parents either the odour of their own or that of foreign nestlings and investigated whether the odour presentation resulted in a change in the number of head saccades, i.e. the rapid horizontal turning of the head, with which birds scan their environment and which can be used as a proxy of arousal. Our experiment indicates that male zebra finches, in contrast to females, differentiate between their own and foreign offspring based on odour cues, as indicated by a significant differences in the change of head saccadic movements between males receiving the own chick odour and males receiving the odour of a foreign chick. Thus, it provides behavioural evidence for olfactory offspring recognition in male zebra finches and also the existence of appropriate phenotypic odour cues of the offspring. The question why females do not show any sign of behavioural response remains open, but it might be likely that females use other signatures for offspring recognition.

## Introduction

In birds, the lack of a detectable behavioural difference against extra pair young or intraspecific brood parasites has led to the conclusion that parents, and particularly males, lack the ability to discriminate against non-related offspring, at least at an early developmental stage of the chicks^1,2^. Whereas nestling discrimination against interspecific brood parasites has been shown in a few species, such as the large-billed Gerygone (*Gerygone magnirostris*) or the superb fairy-wren (*Malurus cyaneus*)^3,4^, evidence for nestling discrimination against intraspecific brood parasites or against extra pair young is lacking. However, the absence of a behavioural differentiation, e.g. the lack of evidence for a preferential feeding of own offspring or for a reduced survival of fostered offspring^1^, does not necessarily imply the absence of offspring recognition. For example, in male Siberian Jay (*Perisoreus infaustus*), aggression towards intruders correlates with the genetic distance between intruder and breeder^5^, indicating that male jays are in general able to assess relatedness of conspecifics. Nevertheless, males did not show a behavioural difference between fostered, i.e. unrelated, and own offspring^5^. Similar results are also found in other birds species, in which individuals recognise kin in certain circumstances, for example during winter flocks^6^, but do not show any sign of kin discrimination in the nest. The apparent lack of discrimination raises the question whether own offspring can not be recognised at the nestling stage. This would be in contrast to other vertebrate taxa, in which mothers recognise their offspring immediately after birth^7–12^, based on olfactory cues. While offspring recognition in mammals is mainly based on olfactory cues, this aspect has been largely ignored as a potential mechanism for birds.

Only recently it has been demonstrated that newly hatched zebra finch hatchlings recognise their parents by olfactory cues^13^, leading to the question whether avian parents, like mammals^14^, are also able to recognise their offspring by smell. Though, the use of olfactory cues has been neglected in this respect, Cohen (1981) demonstrated that parents of ring doves fed artificially scented squabs less frequently than control squabs, resulting in a remarkably lower survival rate of the scented squabs^15^. The author also proved that the reduction in parental care was indeed due to olfactory cues. To our knowledge there is only one other study which investigated whether olfactory cues are used in nestling recognition^16^. In this study, spotless starling (*Sturnus unicolor*) mothers were tested for their ability to recognise their offspring based on olfactory cues. The authors presented females in a cage with two choice chambers their own chick odour and the odour of foreign chick. Females did not show any sign of discrimination ability, neither when the chicks had a closed uropygial gland (chicks age 5–6 days) nor when the chicks had an open uropygial gland (chicks age 12–14 days). Males, however, have not been tested. Here, we investigated olfactory offspring recognition in male and female zebra finches (*Taeniopygia guttata*).

**Figure 1:**
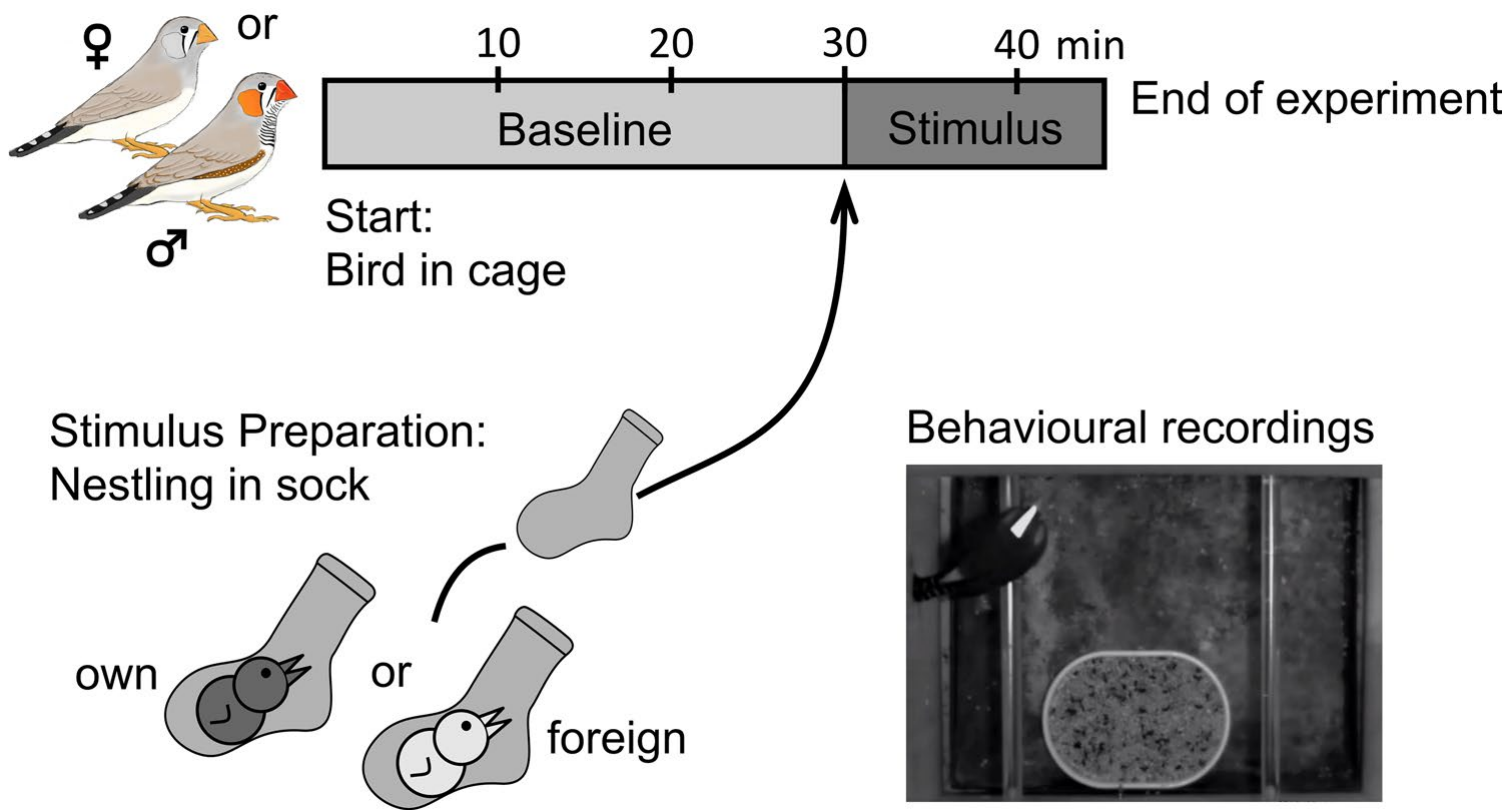
Experimental procedure. At the start of the experiment, test subjects, i.e. either the male or the female, were equipped with an arrow shaped reflectance foil attached to the scalp feathers and were put into the experimental cage. Birds habituated to the test environment for 30 min. Video recordings were taken immediately after the test subjects were put into the cage until 15 min after the stimulus odour was presented, i.e. in total for 45 min. During the time of habituation, i.e. during the first 30 min, we prepared the odour stimuli by putting an own nestling or a foreign nestling into a sock for 30 min. After 30 min the chick was moved to its natal nest and the sock was used as an odour stimulus. For both, baseline and stimulus, we counted the mean number of head saccades within ten consecutive intervals of one minute each and averaged these ten counts to obtain an average number of head saccades per minute.

Zebra finches are colony-breeding songbirds, which form life-long pair bonds and engage in bi-parental brood care^17–19^. The nestling phase of Zebra finch lasts until 17 to 22 days after hatching, followed by a period in which the offspring is still dependent on parental feeding until 35 days of age^19^. Zebra finches have a well-developed sense of smell, and use olfactory cues in various circumstances (reviewed in^20^), for example during mate choice^21^, nest recognition^22,23^, and egg recognition^24^. Furthermore, it has been shown that fledglings prefer the nest odour of their genetic family, even when being raised in a conspecific nest^25^. Hatchlings differentiate between the odour of their parents and unfamiliar adult conspecifics and the preference for the odour of the genetic mother is present even when the chicks hatched in another nest^13^. Both studies show that zebra finches are capable of olfactory kin recognition. Zebra finch parents, as the majority of socially monogamous bird species^26^, experience unrelated offspring, due to intraspecific brood parasitism^27^ or extra-pair paternities^28^, both, however, with rather low levels (Extra pair paternities 5–8% of broods; Intra specific brood parasitism 17.5–36% of broods^26,27^). Whether parents are able to recognise their offspring via smell is, however, unknown. Thus, we examined here whether zebra finch parents differentiate between the odour of their own young and the odour of foreign young (see Fig. 1). To do so, we exposed individual birds either to the odour of their own offspring or to the odour of a foreign conspecific offspring, and measured the change in the individual´s arousal as a response to the specific odour stimulus. As a proxy of arousal we counted the number of head saccades of a bird, as we have done in a previous study^29^. Head saccadic movements are rapid horizontal turnings of the head, with which birds scan their environment, similar to the rapid eye movements in humans. We counted these head saccades, prior and after receiving the stimulus odour, and expected that in case of odour recognition, birds show a change in their arousal, i.e. either increase their head saccadic movements, as a sign of being more aroused, or decrease their movements, as a sign of being less aroused.

## Methods

### Study species and housing conditions

Zebra finches (*Taeniopygia guttata*) are colony-living, monogamous songbirds^17,19^ that are established as one of the most important avian model species^30^. Both parents engage in incubation and feeding the offspring, which are highly dependent on parental care. For the duration of the experiment, each breeding pair, which derived from the population of domesticated zebra finches^31^ at the Bielefeld University, Department of Animal Behaviour, was housed in a two compartment cage (80 × 30 × 40 cm^3^) until the offspring were independent. At the end of the experiments, males as well as females and all offspring were returned to the laboratory stock. Housing conditions are assumed to be superior to natural conditions because all animals had ad libitum food plus additional vitamins, germinated seeds and egg food (CéDé, Evergem, Belgium), given daily once the first offspring hatched. Chickweeds and a water bath were provided once a week. All animals and their offspring were checked daily to verify the health status of the individuals^29^.

### Ethical note

All methods were carried out in accordance with the relevant guidelines and regulations. Housing and breeding of birds were approved by the “Gesundheits-, Veterinär- und Lebensmittelüberwachungsamt der Stadt Bielefeld” (# 530.421630-1, 18.4.2002). Experimental protocols and procedures, involving animals were approved by the LANUV NRW (# 84 02.05.40.17.009). Behavioural tests were conducted in the Department of Animal Behaviour, Bielefeld University. This study was carried out in compliance with the ARRIVE guidelines.

### Odour stimulus preparation

All experiments took place when the brood was on average ten days of age (mean offspring age 9.7 ± 1.48 days). At this time, nestlings have their eyes open and feathers start to grow. The preen gland, considered to be the major source of avian body odours^32–34^, is developing, but not yet functional (personal observations^35^).

We usually used one nestling per nest (N = 14), in cases (N = 6) in which the chicks were much smaller, we used two chicks to guarantee a similar body mass (nestling body mass was on average 8.16 ± 1.44 g) in the sock, and placed them in nylon socks for half an hour to impregnate the material with their body odour, a procedure that has successfully been used in previous studies^13,29,36,37^. As the nestlings were used as a genetic stimulus in one experiment and as the unfamiliar nestling stimulus in another experiment for another adult test bird (see supplement), we kept the same number of nestlings in both socks. We used dark cotton nylon socks (nylon socks, 63% polyamide, 37% cotton, Söckchen Naturelle 60, NUR DIE, DBA Deutschland GmbH, Rheine, Germany). After 30 min, we released the nestlings to their natal nest boxes, e.g. parental cages, and the empty socks, which were impregnated with the nestlings’ odour, were used as odour stimuli. We cleaned the nylon socks with an odourless soap (Eubos, Basispflege, Flüssig Wasch + Dusch, Parfümfrei) after each experiment.

### Experimental setup and procedure

We tested the birds in the same room in which the birds were housed, which guaranteed constant environmental conditions for the individuals. To not influence the outcome of the experiment and to avoid acoustic contact to family members, we brought the mate and the offspring of the experimental bird to an adjacent room for the duration of the test. The experimental setup has been successfully used in a previous study of our lab^29^. Briefly, the experimental cage (28 cm × 20 cm × 51 cm; W × L × H) had two wooden perches 15 cm above the floor and a food cup on the floor. The construction of the experimental cage was similar to the rectangular home cages, with three solid sides made of wood and a cage grid with a door at the front. A rectangular window in the middle of the rear wall led to a wooden nest box (15 × 15 × 15 cm) attached at the outside, filled with coconut fibres and identical to those in the breeding cages. Access to the nest box was prevented by wire mesh. The rear wall of the nest box contained a round hole (diameter 7.5 cm), with a fan (Sunon 40 × 40 × 10 cm (H × L × W), 12 V reduced to 9 V) attached behind. For the test, we placed the odour stimulus sample (empty sock) between the fan and the nest box. The fan emitted a constant air-flow that transported the odour through the nest box into the cage. This setup of providing the odour through a nest box already proved to be successful in odour preference tests^22,24,25,38^. To enable us to count the head saccades, an arrow shaped reflectance foil was attached to the scalp feathers, which could easily be removed when the experiment was finished. We videotaped the behaviour of the bird with a camera (Panasonic WV-BL202-E CCTV Camera) from above, and the recordings were transferred to a computer using an USB drive adapter (Swann 4CH SW 24I-UD4 N3960).

### Experiment procedure

Nineteen zebra finch pairs were used to test whether males and females are able to discriminate their own offspring from unrelated nestlings. Males and females of one pair were tested in randomised order at the same day. In the setup described above, each of the birds was exposed either to the own nestling odour (‘own offspring odour group’, n = 9 males, 9 females) or a conspecific’s nestling’s odour (‘foreign odour group’, n = 10 males, 10 females). To control for potential nest effects, we used a testing protocol based on nest dyads. Each dyad consists of two nests, which were matched by nestling age. This ensured that each nestling was used twice as stimulus, once for an own parent, once as an unfamiliar stimulus for an other adult (see figure in supplement).

For the purpose of the test, each individual was moved to the cage and allowed to habituate to the test environment for 30 min (Fig. 1). A baseline measurement of ten minutes was taken to determine the number of head saccades the bird made without a stimulus immediately prior to the stimulus exposure. During the subsequent presentation of one of the two stimuli, head saccades were analysed five minutes after the odour stimulus presentation begun, similar to a former study^29^. For both, baseline and stimulus, we counted the mean number of head saccades within ten consecutive intervals of one minute each and averaged these ten counts to obtain an average number of head saccades per minute. A head saccade (Hs) was defined as a rapid horizontal turning of the head to either side by more than ten degrees, for details see^29^. The video recordings were used for counting, the observer was blind to the identity of the bird and the type of stimulus provided. To normalise for the differences in activity of individual birds, a proportional rate (in %) of head saccade changes (Hs_Change)  during stimulus presentation was calculated:

$$Hs\_Change = \left( {\frac{\overline{Hs\_stimulus}}{{\overline{Hs\_baseline}}}*100} \right) - 100$$

Positive values indicate an increase in head saccades after the stimulus presentation, whereas negative values represent a decrease.

### Statistical analysis

We used a linear mixed effects model (LMM) based on maximum likelihoods of the package “nlme” by^39^ in R version 3.1.2 with the resulting *Rate of change* as the response variable. *Stimulus type* (own or foreign) and *Parental sex* (male or female) as well as the two-way interaction were included as explanatory variables. Stimulus origin, i.e. the identity of the nestling that was used as stimulus donor, was included as a random effect. However, in a model comparison, the random effect turned out to be non-significant (L-Ratio < 0.001, *p* = 0.999). We found a significant two-way interaction of *Stimulus type* and *Parental sex* and the resulting minimal adequate model was validated by visual inspection of the residuals. As it is not appropriate to interpret the main effects included in a significant interaction^40^, in a second step we performed an analogous analysis for each sex separately, using a linear model (LM).

## Results

Using a linear mixed effect model (LMM), we found a significant interaction of *Stimulus type* (own or foreign) and *Parental sex* (male or female) (LMM: *Stimulus type* x *Parent sex*: T = − 2.38, *p* = 0.03) on the proportion of head saccade change. Thus, the change in arousal towards the odour stimulus was different for males and females. In a following step, we analysed the arousal towards an olfactory, social stimulus separately for males and females.

**Figure 2:**
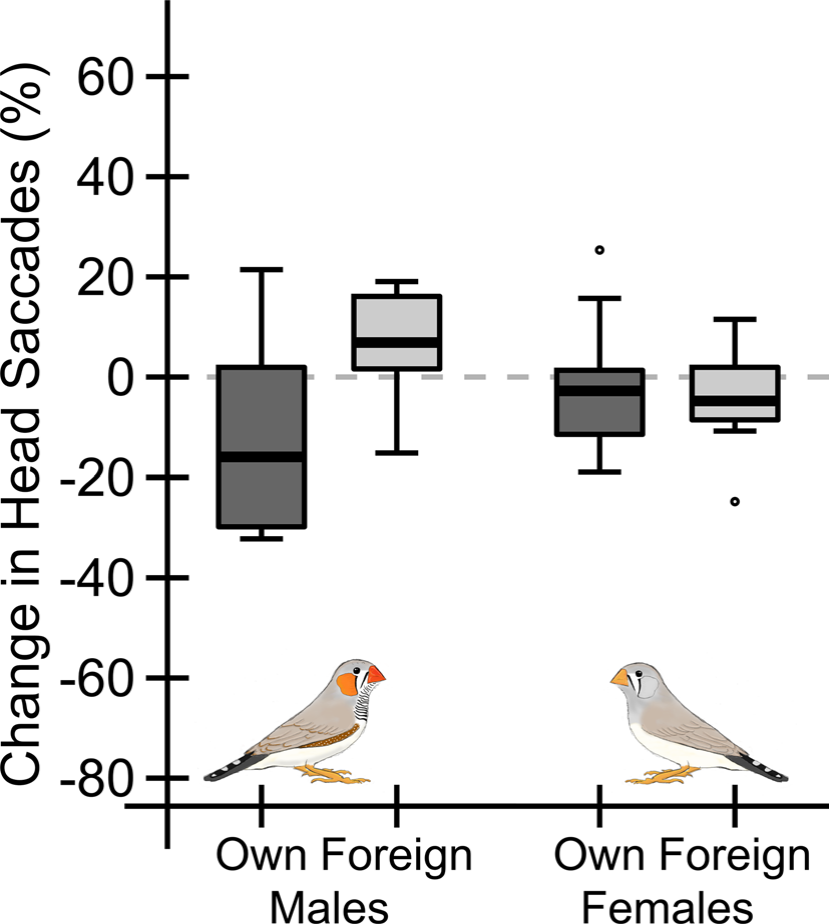
Male and female behavioural reaction, i.e. head saccade change, in response to the different odour stimuli. Shown is the change in head saccades, i.e. the difference in head saccades after odour presentation and before odour presentation. Negative values indicate a decrease in the number of head saccades, whereas positive values indicate an increase. We found a significant interaction of stimulus type and parental sex. Moreover, we found a significant difference in head movements between males receiving the own chick odour and males receiving the foreign chick odour, but not in females.

Females did not show a discrimination between own and foreign offspring, as the proportional change of head saccades in response to the odour stimulus was non-significant (LM: DF = 17, T = − 0.51, *p* = 0.618). In comparison to the baseline measurement, females non-significantly reduced the number of head saccades after the presentation of the own offspring odour (change by − 1.55 ± 14.33%, N = 9, V = 28; *p* = 0.57) and the foreign nestling odour (change by − 4.38 ± 9.31%, N = 10, V = 40.5, *p* = 0.20, Fig. 2).

In contrast to females, we found a significant difference in the proportional change of head saccades in response to the odour stimulus between males receiving the own chick odour and males receiving the foreign chick odour (LM: DF = 17, T = 2.84, *p* = 0.011). Males receiving the own chick odour tended to decrease the number of head saccades (change by − 14.63 ± 17.5%, N = 9, V = 39; *p* = 0.055), whereas males that received the foreign chick odour tended to increase the number of head saccades (change by 6.79 ± 10.4%, N = 10, V = 9, *p* = 0.064, Fig. 2).

## Discussion

In altricial birds, offspring recognition has been thought to be absent at the early nestling stage, at least when tested for visual and acoustic offspring recognition. Olfaction has as yet not been taken into account in this context (except^16^). In spotless starlings, females do not discriminate between the odour of their own offspring and the odour of a foreign offspring and do not preferentially sit in the vicinity of one or the other^16^. Male spotless starlings were not tested, so to our knowledge our study is the first study to test paternal olfactory offspring recognition in a bird. According to our head saccade counts, male zebra finches showed different behavioural responses to the odour of their own offspring and the odour of a foreign offspring. Males that received the own chick odour decreased the amount of head saccades and showed a significantly lower amount of head saccades compared to males that received the foreign chick odour. In contrast, female zebra finches did not show such a behavioural reaction, neither to the own nor to foreign offspring odour. The male response is supported by a previous study in which males were given either a control odour or the odour of their own chick and males decreased the amount of head saccades in response to their own chick odour, while not showing any behavioural differences towards the control^29^. In contrast to earlier assumptions, our finding clearly indicates that zebra finch nestlings carry some phenotypic odour cue that makes the differentiation of own and foreign offspring possible, and it shows that males in contrast to earlier claims are able to recognize these cues.

Males have been said not to discriminate among offspring in their nest^1,41–43^. This lack of observable discrimination between own and foreign offspring within the nest has led to the assumption that the recognition of offspring at an early developmental stage is absent^1,41,44^. However, it is important to note that the absence of a behavioural differentiation does not necessarily imply the absence of offspring recognition. Recent years have already accumulated a number of results showing that zebra finches use odour cues in social contexts (reviewed in^20^). As an example, zebra finch hatchlings are able to recognise their parents scent after hatching, and even their (genetic) mothers odour after being fostered as an egg into a different nest^13^. This suggests an important role of odour for olfactory kin recognition (see also^21,25^).

Admittedly, the present study tested offspring recognition rather than true kin recognition, as all “own” offspring stimulus donors were familiar to the test birds. Thus, there is the possibility that males recognised the familiar scent rather than being able to distinguish between their own offspring and a foreign offspring. However, the differential behavioural reaction of the two male groups (either receiving the own or the foreign chick odour) shows that nestlings differ in their body odours, indicating the existence of a phenotypic cue already at the nestling stage. Based on these results, it would be possible to tackle the question of (true?) kin recognition, using the same method combined with appropriate cross-fostering experiments.

Interestingly, and in contrast to our expectations, females did not show any sign of differentiation between nestlings from the own and from other nests. While our previous studies revealed a well-developed sense of smell in zebra finch females^23,24^, the present experiment showed that the arousal level of the females was similar for both olfactory stimuli, and not significantly different from the baseline level. Females might therefore either be unable to discriminate between own and foreign nestlings, or recognition is indeed possible, but the females did just not show this in our experiment. The first explanation is supported by one other study, where female starlings also failed to show olfactory offspring recognition^16^. However, according to earlier studies on olfactory abilities of zebra finches and blue petrels, females distinguish the odour of their own eggs from that of conspecific eggs^24,45^. Moreover, female zebra finches discriminate between the odours of their own nest and a control nest during the nestling phase of their nestlings^23^. Thus, we can rule out the possibility that females are in general unable to perceive social odours. Another explanation might be that females use a different recognition strategy than males, as it is has been shown for egg recognition in parrotbills^46,47^. Finally, the absence of discrimination might be explained by our small sample size, which might be too small to detect potential discrimination in females.

The pressure for developing offspring recognition is assumed to be higher for colonial than solitary breeding species^48–50^. Zebra finches are colonial birds and indeed, our experiments suggest that male zebra finches show true recognition of their own offspring based on phenotypic cues. This recognition goes beyond contextual cues for recognition, as the observed reaction to the stimulus of the own offspring (i.e., change in arousal) was independent of the nest site and happened in an experimental setting. Males might either imprint on the odour of their own offspring and recognise these even in a different nest, or they may compare their own odour with that of the young and recognise those nestlings that are more similar to the own phenotype. Future research might show whether olfactory cues are also relevant in detecting unrelated offspring within a clutch, for example in cases of extra-pair paternities or conspecific brood parasitism, which are known to occur in zebra finches^27,28,51^.

## Conclusion

Our results show that males receiving the own chick odour behaved significantly different than males receiving the foreign chick odour, implying the ability to recognise offspring based on smell in male zebra finches, whereas no such difference was found in female zebra finches. This indicates the existence of an olfactory phenotypic cue of nestlings and challenges the long-term assumption that male songbirds are unable to recognise their own offspring. These findings underline the impact of odours in social communication in zebra finches and most likely other birds and hopefully inspire more research in this nascent field.

## Supplementary Information

Supplementary Information 1.

Supplementary Information 2.

## Data Availability

All data is published as supplementary material.
