# Supplementary material for: Nestling odour modulates behavioural response in male, but not in female zebra finches
Source: Sci Rep. 2021 Jan 12;11:712. doi: 10.1038/s41598-020-80466-z (PMC7804447; doi:10.1038/s41598-020-80466-z)
Supplement: Supplementary file 2 — Supplementary Information 2. [file 41598_2020_80466_MOESM2_ESM.pdf]

Nestling odour modulates behavioural response in male, but not in female zebra finches

Sarah Golüke[1], Hans-Joachim Bischof[2], Barbara A. Caspers[1]

Supplementary Table 1: Additional information on weight and age of the nestlings, which were used as odour stimuli in the test.

First test date	Natal nest	nestling ID	Weight	Birthdate	Age
21.03.16	EF16	grey1168	7,06	11.03.16	10
21.03.16	EF16	grey1170	6,86	12.03.16	9
21.03.16	EF37	grey1166	8,8	11.03.16	10
21.03.16	EF37	grey1167	7,21	12.03.16	9
22.03.16	EF39	grey1194	8,85	13.03.16	9
22.03.16	EF39	grey1179	6,64	15.03.16	7
22.03.16	EF15	grey1175	5,89	14.03.16	8
22.03.16	EF15	grey1176	4,72	15.03.16	7
25.03.16	EF45	grey1169	11,31	12.03.16	13
25.03.16	EF46	grey1177	8,93	14.03.16	11
27.03.16	EF11	grey1195	9,04	15.03.16	12
27.03.16	EF29	grey1192	9,7	18.03.16	9
28.03.16	EF33	grey1194	9,46	18.03.16	10
31.03.16	EF43	grey1199	9,46	20.03.16	11
31.03.16	EF20	grey1201	7,21	22.03.16	9
01.04.16	EF5	grey1205	6,73	23.03.16	9
01.04.16	EF40	grey1206	7,3	23.03.16	9
02.04.16	EF56	grey1207	9,35	23.03.16	10
02.04.16	EF56	grey1214	7,46	25.03.16	8
02.04.16	EF53	grey1208	9,06	23.03.16	10
02.04.16	EF53	grey1209	8,44	23.03.16	10
05.04.16	EF58	grey1216	9,53	25.03.16	11
05.04.16	EF26	grey1231	6,71	27.03.16	9
06.04.16	EF52	grey1233	7,79	28.03.16	9
08.04.16	EF47	grey1232	8,82	27.03.16	12
08.04.16	EF48	grey1225	10,03	27.03.16	12

Mean	8,17	Mean	9,73
St. deviation	1,46	St. deviation	1,48

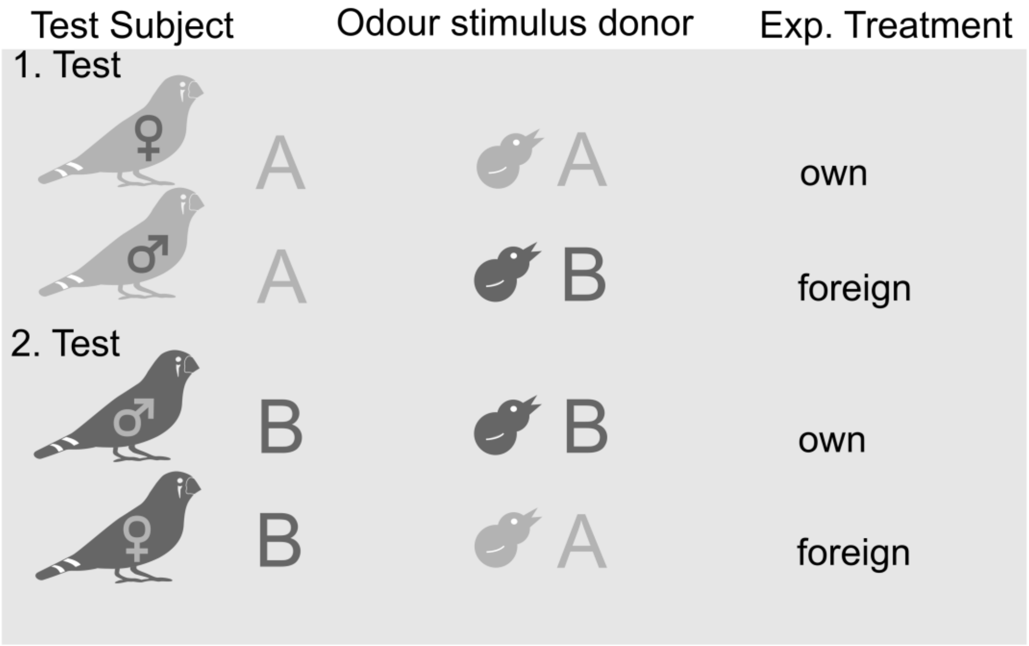

Supplementary Figure 1: Exemplary testing scheme. The testing protocol is based on nest dyads. Each dyad consists of two nests, which we matched by nestling age. This ensured that each nestling was used twice as stimulus, once for an own parent, once as an unfamiliar stimulus for another adult, e.g. nestling A was used as an own stimulus for its genetic mother and as an unfamiliar stimulus for the female of nest B. This way, the testing protocol controls for potential age and weight effects of the offspring. We alternated the order of males and females as well as the stimuli presented.

Supplementary Table 2: Means and standard deviations of the number of head saccades (#) within one minute,.

		Baseline	Own Offspring	Foreign Offspring
Females	n	19	9	10
	Mean	103,36	94,38	103,48
	St. deviation	17,44	16,09	10,96
Males	n	19	9	10
	Mean	100,46	86,75	105,58
	St. deviation	16,67	19,32	15,86
